# *Health Equity* Journal: Special Issue Guest Editorial

**DOI:** 10.1089/heq.2023.29038.mmo

**Published:** 2023-11-30

**Authors:** Michelle Morse, Adriana Joseph, Chandra Ford, Ruqaiijah Yearby, Nichola Davis

**Affiliations:** ^1^ Chief Medical Officer, Deputy Commissioner, CHECW, New York City Department of Health and Mental Hygiene (DOHMH), New York, New York, USA.; ^2^ New York City Department of Health and Mental Hygiene (DOHMH), New York, New York, USA.; ^3^ Professor, Department of Community Health Sciences, Founding Director, Center for the Study of Racism, Social Justice & Health, UCLA Fielding School of Public Health, Los Angeles, California, USA.; ^4^ Kara J. Trott Professor of Law, Moritz College of Law, The Ohio State University, Columbus, Ohio, USA.; ^5^ Vice President, Chief Population Health Officer, NYC Health+Hospitals (NYC H+H), New York, New York, USA.

The practices of contemporary clinical decision-making and care rely heavily on racial biological essentialism, which is a set of ideas originating in modern science that describes populations as comprising distinct subpopulations with unique sets of essential, heritable characteristics and propensities (i.e., races) purportedly due to their biology.^1^ Racial biological essentialism exaggerates the relevance of biology to health inequities and promotes the misuse of race (e.g., “Black race”) in clinical decision-making, care, and research. Decades of research and scholarship^2–4^ (e.g., human genome project which inadvertently established that more genetic variation exists within each U.S. racial category than between them) have shown that race is fundamentally not biological. A substantial body of evidence clarifies that race is a sociopolitical, not biological construct.^5^ Nevertheless, the harmful, unscientific practices of racial biological essentialism persist, which helps explain why the misuse of race in clinical decision-making, research, and education remains pervasive.

For many years, medical trainees, health equity scholars, and public health physicians have explained how race consciousness (i.e., racism consciousness), which is the understanding of race as a sociopolitical construct, provides a more useful understanding for medicine and public health than racial biological essentialism does.^6^ This work has taken many forms, including the de-implementation of race-based algorithms used as clinical decision-making tools. In 2020, the Ways and Means Committee in the U.S. House of Representatives asked professional societies across medical disciplines to rethink their use of race-based clinical algorithms. The Committee sent a “Request for Information” (RFI) to medical professional societies endorsing the elimination of race-based clinical algorithms. The study findings and RFI responses were captured in a 2021 report and captured responses from the professional societies as well as recommendations to improve clinical decision-making.^7^ The Agency for Healthcare Research and Quality (AHRQ) is also taking on this issue. At the time of this publication, AHRQ is undertaking a systematic review to provide Congress and the public responses to key questions on the impact of race-based clinical algorithms on health outcomes, and what can be done to address and/or mitigate racial bias on the development, validation, etc., of clinical algorithms.^8^

Since 2021, several professional societies have updated their positions on the inclusion of race in clinical algorithms within their respective specialties. The National Kidney Foundation and the American Society of Nephrology officially endorsed an estimated glomerular filtration rate (e-GFR) calculator without a race variable.^9^ The American College of Obstetrics and Gynecology no longer endorses a vaginal birth after caesarean calculator that uses race.^10^ Most recently, the American Thoracic Society issued updated recommendations in spirometry testing and the race-neutral reference equation for all patients, irrespective of race.^11^

As research continues to elucidate the harms and any benefits from including race in clinical algorithms, the urgency to address race-based algorithms is only intensifying. For instance, 35% of Americans suffering from renal failure are Black, while only representing 13% of the population.^12^ Many social and clinical factors contribute to this stark inequity, including the misuse of race to modify the e-GFR score, which is used in the diagnosis and treatment of chronic kidney disease (CKD). Race and other social factors have been linked to the e-GFR and other statistics, which has been associated with disproportionate suffering due to (CKD) and its sequalae among Black populations (e.g., higher rates of end-stage CKD diagnosis and lower rates of kidney transplantation eligibility among Black populations).^13^

Health equity experts agree to the implementation of nonrace-based clinical algorithms that cannot be subjected to the over 10-year timeframe typical for medical research and its adoption into practice.^14^ To meet the urgency of this moment, the NYC Department of Health and Mental Hygiene launched the Coalition to End Racism in Clinical Algorithms (CERCA). This coalition is a citywide initiative consisting of both safety-net hospitals and academic medical centers representing all five boroughs of NYC. Participation in CERCA requires that each coalition member commit to de-implement at least one race-based algorithm. Members are also required to furnish work, evaluation, and patient engagement plans regarding their de-implementation of race-based algorithms.^15,16^

In the summer of 2023, the NYC Department of Health and Mental Hygiene hosted the first annual New York City Anti-racism in Medical Education Symposium in partnership with the Josiah Macy Jr. Foundation, the American Academy of Medical Colleges, and the Fund for Public Health NYC. This symposium aimed to identify key stakeholders involved in anti-racism and curriculum development at NYC medical schools and understand the depth and breadth of anti-racism praxis incorporated into their educational programming.^16^

This special issue of *Health Equity* hopes to contribute to this growing body of knowledge regarding the de-implementation efforts needed to holistically address and eradicate race essentialism from practice and education. Specifically, this issue highlights scholarship in the following areas:

- Historical origins of race adjustment in medicine, clinical decision-making tools, and artificial intelligence tools in medicine;
- Current activities, successes, and challenges around removal of race from clinical decision-making tools at the institution- and system-level and its impact on patient outcomes;
- City, state, and federal policies and policy analysis supporting removal of race adjustment from clinical decision-making tools; and
- Programs, interventions, and policies intended to interrupt or end algorithmic racial discrimination in medicine and health care.

We hope this publication captures the latest work in addressing race-based medicine and facilitate the adaptation and implementation of initiatives to correct and mitigate the harmful effects of racial discrimination in health care. As we chart the next steps of this movement—which include equitable transplantation access, federal changes in Medicaid and Medicare policy on use of race-based algorithms, biases in artificial intelligence, application of public health critical race praxis (PHCRP) in research, to name a few emerging areas—remaining abreast of current and needed work will be essential to realizing a more equitable, just, and healthy society.

According to PHCRP, which is a health equity offshoot of Critical Race Theory, the first step toward advancing health equity is to acknowledge how the conventions of our field help reinforce inequities however well-intentioned our efforts may be. The use of arbitrary race corrections in clinical algorithms relies on and reifies racial biological determinism. It undermines the ability of clinicians to uphold their commitment to beneficence, nonmaleficence, and justice in the provision of care. Failure to uphold them harms minoritized patients and communities through, for instance, delayed or missed diagnoses and the exacerbation of racialized stigmata. A substantial body of scholarship and research now exists to promote more equitable clinical decision-making and care. Consistent with the PHCRP principle of *disciplinary self-critique*, this special issue documents the continued misuse of race in clinical algorithms. It also offers constructive alternatives that can be implemented immediately.^17,18^

## Abbreviations Used

AHRQ: Agency for Healthcare Research and Quality
CERCA: Coalition to End Racism in Clinical Algorithms
CKD: chronic kidney disease
e-GFR: estimated glomerular filtration rate
PHCRP: public health critical race praxis
RFI: request for information
